# Expression of CD73 and VEGF in salivary gland carcinomas: associations with clinicopathological characteristics in Vietnamese population

**DOI:** 10.1186/s12885-025-15129-1

**Published:** 2025-10-31

**Authors:** Tuan Duc Nguyen, Hong Thi Nguyen, Chau Giang Huynh, Truc Thanh Thai, Truong Xuan Bui, Tu Anh Thai, Tu Thanh Duong, Minh Duc Nguyen, Dung Anh Vo, Uy Van Pham, Dung Huynh Thi Nguyen, Anh Nguyet Thi Nguyen

**Affiliations:** 1 Ho Chi Minh City Odonto-Maxillo Facial Hospital, Ho Chi Minh City, Vietnam; 2 Department of Oral Medicine and General Dentistry, Nguyen Tat Thanh University, Ho Chi Minh City, Vietnam; 3 Department of Pathology, Hung Vuong Hospital, Ho Chi Minh City, Vietnam; 4 Department of Medical Statistics and Informatics, University of Medicine and Pharmacy at Ho Chi Minh City, Ho Chi Minh City, Vietnam; 5 Ho Chi Minh City Oncology Hospital, Ho Chi Minh City, Vietnam; 6 Department of Pathology, University of Medicine and Pharmacy at Ho Chi Minh City, Ho Chi Minh City, Vietnam; 7 Department of Oralpathology, Faculty of Odonto-Stomatology, University of Medicine and Pharmacy at Ho Chi Minh City, Ho Chi Minh City, 700000 Vietnam

**Keywords:** Salivary gland carcinoma, CD73, VEGF, Ki-67, Immunohistochemistry, Biomarker, Vietnam, Prognosis, Personalized medicine

## Abstract

**Background:**

Salivary gland carcinoma is a rare malignancy with diverse histological subtypes and poor prognosis, particularly when diagnosed at advanced stages. Recent evidence suggests that biomarkers such as CD73 and VEGF may play important roles in tumor progression and immune evasion, yet limited studies have evaluated their expression and clinical significance in Southeast Asian populations. This study aimed to determine the expression rates of CD73 and VEGF in salivary gland carcinoma and identify clinicopathological factors associated with their expression in a Vietnamese patient cohort.

**Methods:**

A retrospective study was conducted on 111 salivary gland carcinoma patients who underwent surgical treatment in Ho Chi Minh City, Vietnam. Immunohistochemical analysis of CD73, VEGF, and Ki-67 expression was performed on paraffin-embedded tumor samples. Logistic regression models were used to identify factors independently associated with biomarker expression.

**Results:**

CD73 and VEGF were expressed in 53.2% and 66.7% of salivary gland carcinoma cases, respectively. CD73 expression was significantly associated with female gender (OR = 2.74, 95% CI 1.07–7.01), tumor stage T2 (OR = 6.59, 95% CI 1.36–31.90) and T4 (OR = 9.13, 95% CI 1.68–49.66), mucoepidermoid carcinoma subtype (OR = 10.62, 95% CI 2.77–40.69), and higher Ki-67 levels (OR = 3.61 per 10% increase, 95% CI 1.26–10.31). In contrast, lymph node level N2 was inversely associated with CD73 (OR = 0.08, 95% CI 0.01–0.70). VEGF expression was independently more likely in patients with normal body mass index compared to those who were overweight or obese (OR = 0.30 for overweight/obese vs. normal, 95% CI 0.12–0.78), and was also associated with increased Ki-67 expression (OR = 2.95, 95% CI 1.09–7.99).

**Conclusions:**

High expression rates of CD73 and VEGF suggest their potential as prognostic biomarkers and therapeutic targets in salivary gland carcinoma. Their associations with tumor stage, histology, BMI, and proliferation index (Ki-67) highlight the need for further research on personalized treatments. These findings provide important insights into biomarker expression in Vietnamese salivary gland carcinoma patients and lay the groundwork for biomarker-driven therapeutic strategies.

**Supplementary Information:**

The online version contains supplementary material available at 10.1186/s12885-025-15129-1.

## Background

Salivary gland carcinoma (SGC) is a rare cancer, accounting for approximately 3–5% of head and neck malignancies [1] and only about 0.5% of all cancers [2]. In Vietnam, the age-standardized incidence of SGC is 0.32 per 100,000 people [3]. This type of cancer often presents a high mortality rate and a poor prognosis if not detected and treated early. Most cases of SGC are diagnosed at advanced stages due to atypical clinical symptoms and the diversity of histological subtypes. Untreated, SGC can metastasize to the lungs, bones, and other organs, significantly affecting patients’ quality of life and resulting in a 5-year survival rate ranging from 72% to 85% [4–6].

The development of biomarkers for cancer diagnosis and prognosis has improved treatment effectiveness, with CD73 and VEGF emerging as potential biomarkers for SGC. CD73, an enzyme involved in immune regulation through the adenosine pathway, has been associated with cancer cell immune evasion [7, 8]. VEGF plays a key role in angiogenesis, supplying nutrients to tumors and thereby promoting cancer cell growth and metastasis [8]. These two proteins are closely related. Through the adenosine pathway, CD73 can stimulate the production and secretion of VEGF from tumor cells [8, 9]. This VEGF, together with the extracellular VEGF pool, leads to a more potent overall effect of VEGF. While several international studies have explored the roles of CD73 and VEGF in various cancers, their application in SGC remains limited, with insufficient evidence, particularly regarding their prognostic and therapeutic implications.

Several studies have found that the overexpression of CD73 [8, 10] and VEGF [11, 12] is associated with invasion status, malignancy grade, lymph node metastasis, and poor prognosis in SGC. Recently, CD73 inhibition therapy has also been undergoing clinical trials for cancer treatment, showing some promising results [7]. Furthermore, anti-angiogenic therapies targeting VEGF have been developed, such as Avastin (Bevacizumab). Avastin is the first cancer treatment drug that inhibits the formation of blood vessels supplying tumors and was approved for use by the U.S. Food and Drug Administration (FDA) in 2004 [13]. Avastin is indicated for the treatment of advanced head and neck cancers or cases where traditional treatment methods have failed. Therefore, understanding the rate of expression of these biomarkers and their associated factors is crucial so that early intervention can be made to optimize treatment for patients with SGC.

Currently, research on SGC in Vietnam remains limited, with most studies focusing on epidemiological, clinical factors, and some other immune markers such as Ki-67 and Her2 [14, 15]. No detailed research has yet explored the roles of biomarkers like CD73 and VEGF in SGC, even though these may be important factors in assessing prognosis and personalizing treatment. Based on these existing gaps, this study aims to determine the expression levels of the CD73 and VEGF biomarkers in SGC and their associated factors in Vietnamese population.

## Methods

### Settings and participants

This study was from December 21 st, 2017, to December 31 st, 2022, at the Department of Pathology, University of Medicine and Pharmacy at Ho Chi Minh City, and the Pathology Department at Ho Chi Minh City Oncology Hospital. All 147 patients diagnosed with SGC who underwent surgical treatment at Ho Chi Minh City Oncology Hospital, with specimens available for immunohistochemical analysis from January 1 st, 2016, to 31 st, December 2017, were recruited. Patients were included if they had complete medical records, a confirmed histopathological diagnosis of SGC, and paraffin-embedded tissue blocks (FFPE) suitable for immunohistochemical staining. We excluded patients with recurrent SGC or insufficient tissue samples for analysis. Additionally, cases with insufficient number of tumor cells on immunohistochemistry slides were excluded from the data analysis, as the study focused on several biomarkers.

### Procedures and measurements

Patient samples were collected from SGC specimens without prior treatments such as chemotherapy or radiotherapy. Histopathological information, including tumor type and stage, along with immunohistochemical data for biomarkers Ki-67, CD73, and VEGF were extracted from clinical records. Hematoxylin and eosin (H&E) stained slides were used for initial histopathological evaluation. If the H&E slides were of insufficient quality, new slides were prepared from FFPE. The H&E slides were reviewed and the histopathological diagnoses were confirmed based on the 2017 WHO classification [16].

We used the following antibodies: Ki-67 Antigen (MIB-1, M7240, DAKO, dilution 1:100), VEGF (C-1, SC-7269, Santa Cruz Biotechnology, dilution 1:200), and CD73 (D7F9A 13160, Cell Signaling Technology, dilution 1:200). Tissue microarray was performed using the Tissue Microarray kit from Unitma (Korea). Immunohistochemical staining was carried out using the automated immunostainer Ventana (Roche, USA). Tonsil tissue was used as the positive control. Several studies have used different cutoff thresholds for K-i67 in salivary gland cancer, ranging from 5% to 30% [17–19]. In our study, Ki-67 was assessed based on the percentage of tumor cell nuclei staining positive, with high expression defined based on a cutoff of ≥ 10%, which indicated aggressive tumor behavior. According to research by Bauer et al. [8], CD73 expression was determined as positive when there was strong membranous staining in over 1% of tumor cells, reflecting potential immune escape mechanisms. In Park’s study, the authors employed a semi-quantitative scoring system [19]. Based on this system, a case would be considered VEGF-positive when approximately 50% of tumor cells expressed the marker. In our study, VEGF expression was considered positive if there was intense cytoplasmic staining in more than 50% of the tumor cells.

For evaluation of CD73, VEGF, and Ki-67, immunohistochemical staining was conducted on 4 to 5 μm thick tissue sections using standardized protocols. Ki-67 was quantified as a proliferation index, calculated by the percentage of positively stained tumor cell nuclei over the total number of tumor cells observed. CD73 and VEGF levels were assessed semi-quantitatively based on staining intensity and distribution within tumor cells.

Two trained pathologists independently evaluated the H&E and immunohistochemical slides to ensure consistency. In cases where the two pathologists disagreed on interpretation, a third senior pathologist reviewed the slides and provided the final assessment. To assess the level of agreement between two experts in evaluating the immunohistochemical staining results based on the expression level (quantitative), we used the Intraclass Correlation Coefficient (ICC). The agreement was very high and statistically significant, with an ICC of 0.91 (95% CI: 0.87–0.94; *p* < 0.001). To evaluate the agreement when the staining results were interpreted as high or low expression (qualitative), we used the Kappa statistic. The agreement was also very high (87.2%), with a Kappa value of 0.74, which was statistically significant (*p* < 0.001).

### Data analysis

All data were analyzed using Stata version 17. The Kolmogorov-Smirnov test was employed to determine whether quantitative variables followed a normal distribution. Categorical variables were compared using Chi-square (χ²) tests or Fisher’s Exact tests as appropriate to analyze percentage differences. For continuous variables, the Student’s t-test was used for comparing means in normally distributed data, while the Mann-Whitney U and Kruskal-Wallis tests were applied for non-normally distributed data. Univariate logistic regression was used to calculate odds ratios (ORs) and 95% confidence intervals (CIs) to examine the association between clinicopathological characteristics and the expression of CD73 and VEGF. Variable selection for multiple logistic regression was based on a p-value < 0.2 in the univariate analysis and on literature review. Steps used to fit the final logistic regression model were based on guideline from Hosmer et al. [20]. Model fit was assessed through the Pearson Chi-squared goodness-of-fit test, the Hosmer-Lemeshow test and the area under the curve (AUC). All tests were two-sided, with a significance level set at *p* < 0.05.

## Results

### Patient’s characteristics

**Fig. 1:**
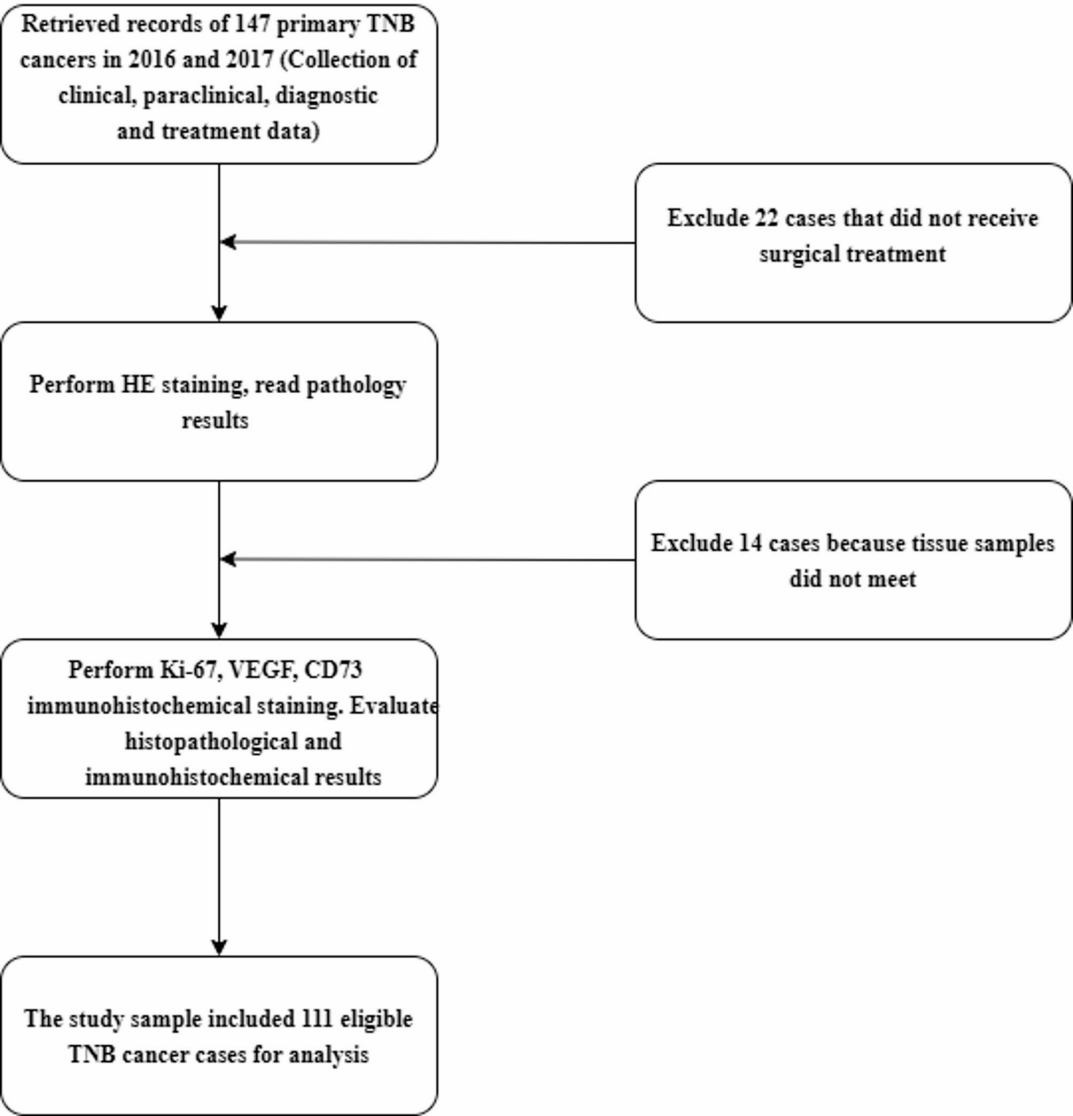
Flow chart of patient selection

Among the 111 patients with SGC included in data analysis (Fig. 1), the mean age was 47.1 (SD = 17.3) years. More than one third (34.2%) of patients with SGC were less than 40 years old. Most patients (60.4%) were female. Regarding body mass index (BMI), the majority fell within the normal range (55.0%), while 11.7% were underweight and 33.3% were classified as overweight or obese. Major salivary glands, particularly the parotid gland, were the most common sites of cancer, accounting for 77.5% of cases, with the remaining 22.5% involving minor salivary glands (Table 1).

**Table 1:** Clinical characteristics associated with CD73 and VEGF expression

Characteristics		CD73 Expression		*p*	OR (95% CI)	VEGF Expression		*p*	OR (95% CI)
	Total (*n* = 111)	Yes (*n*= 59, 53.2%)	No (*n* = 52, 46.9%)			Yes (*n* = 74, 66.7%)	No (*n* = 37, 33.3%)		
Age (years), Mean (SD)	47.1 (17.3)	47.7 (18.2)	46.4 (16.5)	0.701	1.00 (0.98–1.03)	46.5 (16.3)	48.2 (19.5)	0.628	0.99 (0.97–1.02)
Age category (years)									
<40	38 (34.2)	19 (50.0)	19 (50.0)	0.751	1	26 (68.4)	12 (31.6)	0.953	1
40–59	46 (41.4)	24 (52.2)	22 (47.8)		1.09 (0.46–2.58)	30 (65.2)	16 (34.8)		0.87 (0.35–2.16)
≥60	27 (24.3)	16 (59.3)	11 (40.7)		1.45 (0.54–3.94)	18 (66.7)	9 (33.3)		0.92 (0.32–2.64)
Gender									
Male	44 (39.6)	19 (43.2)	25 (56.8)	0.088	0.51 (0.24–1.11)	27 (61.4)	17 (38.6)	0.337	0.68 (0.30–1.51)
Female	67 (60.4)	40 (59.7)	27 (40.3)		1	47 (70.1)	20 (29.9)		1
BMI (kg/m^2^), Mean (SD)	22.0 (3.3)	21.7 (3.4)	22.4 (3.1)	0.291	0.94 (0.84–1.05)	21.9 (3.2)	22.3 (3.5)	0.503	0.96 (0.85–1.08)
BMI category (kg/m^2^)									
Underweight	13 (11.7)	9 (69.2)	4 (30.8)	0.447	1	7 (53.8)	6 (46.2)	0.037	1
Normal	61 (55.0)	30 (49.2)	31 (50.8)		0.43 (0.12–1.55)	47 (77.0)	14 (23.0)		2.88 (0.83–9.97)
Overweight/Obese	37 (33.3)	20 (54.1)	17 (45.9)		0.52 (0.14–2.00)	20 (54.1)	17 (45.9)		1.01 (0.28–3.58)
Salivary gland									
Major salivary gland	86 (77.5)	44 (51.2)	42 (48.8)	0.436	0.70 (0.28–1.73)	55 (64.0)	31 (36.0)	0.261	0.56 (0.20–1.55)
Minor salivary glands	25 (22.5)	15 (60.0)	10 (40.0)		1	19 (76.0)	6 (24.0)		1
Clear margins									
Yes	58 (52.3)	33 (56.9)	25 (43.1)	0.408	1.37 (0.65–2.90)	35 (60.3)	23 (39.7)	0.139	0.55 (0.24–1.22)
No	53 (47.7)	26 (49.1)	27 (50.9)		1	39 (73.6)	14 (26.4)		1
Mobility									
Fixed	43 (38.7)	24 (55.8)	19 (44.2)	0.581	1	31 (72.1)	12 (27.9)	0.513	1
Poor	25 (22.5)	11 (44.0)	14 (56.0)		0.62 (0.23–1.68)	17 (68.0)	8 (32.0)		0.82 (0.28–2.40)
Mobile	43 (38.7)	24 (55.8)	19 (44.2)		1.00 (0.43–2.34)	26 (60.5)	17 (39.5)		0.59 (0.24–1.46)
Any invasion									
Yes	56 (50.5)	32 (57.1)	24 (42.9)	0.395	1.38 (0.65–2.92)	40 (71.4)	16 (28.6)	0.283	1.54 (0.70–3.42)
No	55 (49.5)	27 (49.1)	28 (50.9)		1	34 (61.8)	21 (38.2)		1
Nerve Invasion									
Yes	29 (26.1)	17 (58.6)	12 (41.4)	0.492	1.35 (0.57–3.18)	20 (69.0)	9 (31.0)	0.760	1.15 (0.46–2.86)
No	82 (73.9)	42 (51.2)	40 (48.8)		1	54 (65.9)	28 (34.1)		1
Muscle Invasion									
Yes	21 (18.9)	11 (52.4)	10 (47.6)	0.937	0.96 (0.37–2.49)	15 (71.4)	6 (28.6)	0.607	1.31 (0.46–3.72)
No	90 (81.1)	48 (53.3)	42 (46.7)		1	59 (65.6)	31 (34.4)		1
Bone Invasion									
Yes	21 (18.9)	10 (47.6)	11 (52.4)	0.572	0.76 (0.29–1.97)	16 (76.2)	5 (23.8)	0.304	1.77 (0.59–5.27)
No	90 (81.1)	49 (54.4)	41 (45.6)		1	58 (64.4)	32 (35.6)		1
Skin Invasion									
Yes	7 (6.3)	3 (42.9)	4 (57.1)	0.704	0.64 (0.14–3.02)	5 (71.4)	2 (28.6)	0.999	1.27 (0.23–6.87)
No	104 (93.7)	56 (53.8)	48 (46.2)		1	69 (66.3)	35 (33.7)		1

### Clinical characteristics and CD73, VEGF expression

The expression rate of CD73 biomarkers was found to be 53.2% (illustrated in Appendix 1). Female patients showed a slightly higher rate of CD73 expression (59.7%) compared to males (43.2%) (*p* = 0.088). A higher rate of CD73 expression was observed in patients with underweight BMI (69.2%) and overweight/obese (54.1%) compared to normal BMI (49.2%) (*p* = 0.447). However, these differences were not statistically significant. By gland type, minor salivary gland cancers had a higher percentage of CD73 expression (60.0%) compared to major salivary glands (51.2%) (*p* = 0.436). Tumor margins and mobility were not significantly associated with CD73 expression. Specifically, there was no significant difference in CD73 expression between tumors with well-defined and ill-defined margins, nor among groups with different levels of mobility (Table 1).

The expression rate of VEGF biomarkers was found to be 66.7% (illustrated in Apendix 1). The presence of VEGF expression tended to be higher in females (70.1%) than males (61.4%), but this difference was not statistically significant (*p* = 0.337). However, the VEGF expression rate was significantly lower in patients who were underweight (53.8%) or overweight and obese (54.1%) than in those with normal BMI (77.0%) (*p* = 0.037). More than three-fourths (76.0%) of patients with SGC involving minor salivary glands had VEGF expression, compared to 64% of those with major salivary glands (*p* = 0.261) (Table 1).

### Clinicopathological characteristics and CD73, VEGF expression

The study identified seven types of salivary gland carcinoma. The most common was mucoepidermoid carcinoma, followed by adenoid cystic carcinoma (Appendix 2). Primary tumors classified as T1 and T2 accounted for 41.4% of cases, with 10.8% of patients having T1 tumors and 30.6% having T2 tumors. Advanced primary tumors (T3 and T4) were observed in 58.5% of patients, with T4 cases comprising 40.5% of the sample. Lymph node involvement was relatively low, with only 17.1% of patients presenting with positive lymph node status (N1 or N2), while 82.9% had no regional lymph node metastasis (N0). Distant metastasis (M1) was rare, appearing in only 2.7% of patients. Most cases were classified in advanced stages (S3 and S4), with stage S4 making up 44.1% of the cases. Histologically, mucoepidermoid carcinoma (MEC) was the most common type (53.2%), followed by adenoid cystic carcinoma (23.4%), while high-grade tumors were more frequent, representing 65.8% of the sample (Table 2).

**Table 2:** Clinicopathological characteristics associated with CD73 and VEGF expression

Characteristics	CD73 Expression			*p*	OR (95% CI)	VEGF Expression		*p*	OR (95% CI)
	Total (*n* = 111)	Yes (*n* = 59, 53.2%)	No (*n* = 52, 46.9%)			Yes (*n* = 74, 66.7%)	No (*n* = 37, 33.3%)		
Primary tumor (T)									
T1	12 (10.8)	3 (25.0)	9 (75.0)	0.062	1	10 (83.3)	2 (16.7)	0.350	1
T2	34 (30.6)	22 (64.7)	12 (35.3)		5.50 (1.25–24.26)	19 (55.9)	15 (44.1)		0.25 (0.05–1.34)
T3	20 (18.0)	8 (40.0)	12 (60.0)		2.00 (0.41–9.74)	14 (70.0)	6 (30.0)		0.47 (0.08–2.81)
T4	45 (40.5)	26 (57.8)	19 (42.2)		4.11 (0.98–17.23)	31 (68.9)	14 (31.1)		0.44 (0.09–2.29)
Regional lymph node (N)									
N0	92 (82.9)	49 (53.3)	43 (46.7)	0.740	1	60 (65.2)	32 (34.8)	0.843	1
N1	6 (5.4)	4 (66.7)	2 (33.3)		1.76 (0.31–10.06)	4 (66.7)	2 (33.3)		1.07 (0.19–6.14)
N2	13 (11.7)	6 (46.2)	7 (53.8)		0.75 (0.23–2.41)	10 (76.9)	3 (23.1)		1.78 (0.46–6.92)
Distant Metastasis (M)									
M1	3 (2.7)	2 (66.7)	1 (33.3)	0.999	1.79 (0.16–20.33)	2 (66.7)	1 (33.3)	0.999	1.00 (0.09–11.40)
M0	108 (97.3)	57 (52.8)	51 (47.2)		1	72 (66.7)	36 (33.3)		1
Stage (S)									
S1	12 (10.8)	3 (25.0)	9 (75.0)	0.134	1	10 (83.3)	2 (16.7)	0.262	1
S2	33 (29.7)	21 (63.6)	12 (36.4)		5.25 (1.19–23.22)	18 (54.5)	15 (45.5)		0.24 (0.05–1.27)
S3	17 (15.3)	8 (47.1)	9 (52.9)		2.67 (0.53–13.43)	11 (64.7)	6 (35.3)		0.37 (0.06–2.25)
S4	49 (44.1)	27 (55.1)	22 (44.9)		3.68 (0.89–15.27)	35 (71.4)	14 (28.6)		0.50 (0.10–2.58)
Histological Type									
Mucoepidermoid carcinoma	59 (53.2)	38 (64.4)	21 (35.6)	0.006	1	36 (61.0)	23 (39.0)	0.405	1
Adenoid cystic carcinoma	26 (23.4)	7 (26.9)	19 (73.1)		0.20 (0.07–0.56)	19 (73.1)	7 (26.9)		1.73 (0.63–4.77)
Others	26 (23.4)	14 (53.8)	12 (46.2)		0.64 (0.25–1.65)	19 (73.1)	7 (26.9)		1.73 (0.63–4.77)
Histological grade									
High grade	73 (65.8)	35 (47.9)	38 (52.1)	0.128	0.54 (0.24–1.20)	47 (64.4)	26 (35.6)	0.479	0.74 (0.32–1.72)
Low grade	38 (34.2)	24 (63.2)	14 (36.8)		1	27 (71.1)	11 (28.9)		1
Ki-67, Mean (SD)	7.6 (12.1)	10.1 (15.6)	4.8 (4.9)	0.014	1.06 (1.00–1.12)	9.4 (14.1)	4.1 (5.0)	0.004	1.09 (1.00–1.18)

The analysis of CD73 and VEGF expression in relation to these characteristics revealed some significant associations. CD73 expression was notably higher in T2 tumors, with an odds ratio (OR) of 5.50 (95% CI 1.25–24.26), indicating increased expression in these cases compared to T1 tumors. MEC showed significantly elevated CD73 expression (*p* = 0.006), while adenoid cystic carcinoma displayed lower CD73 expression. In terms of VEGF, no significant association was noted with tumor stage, lymph node status, or histological grade. Additionally, Ki-67 levels were significantly associated with both CD73 and VEGF expression (*p* = 0.014 and *p* = 0.004, respectively) (Table 2).

### Factors independently associated with CD73, VEGF expression

**Fig. 2:**
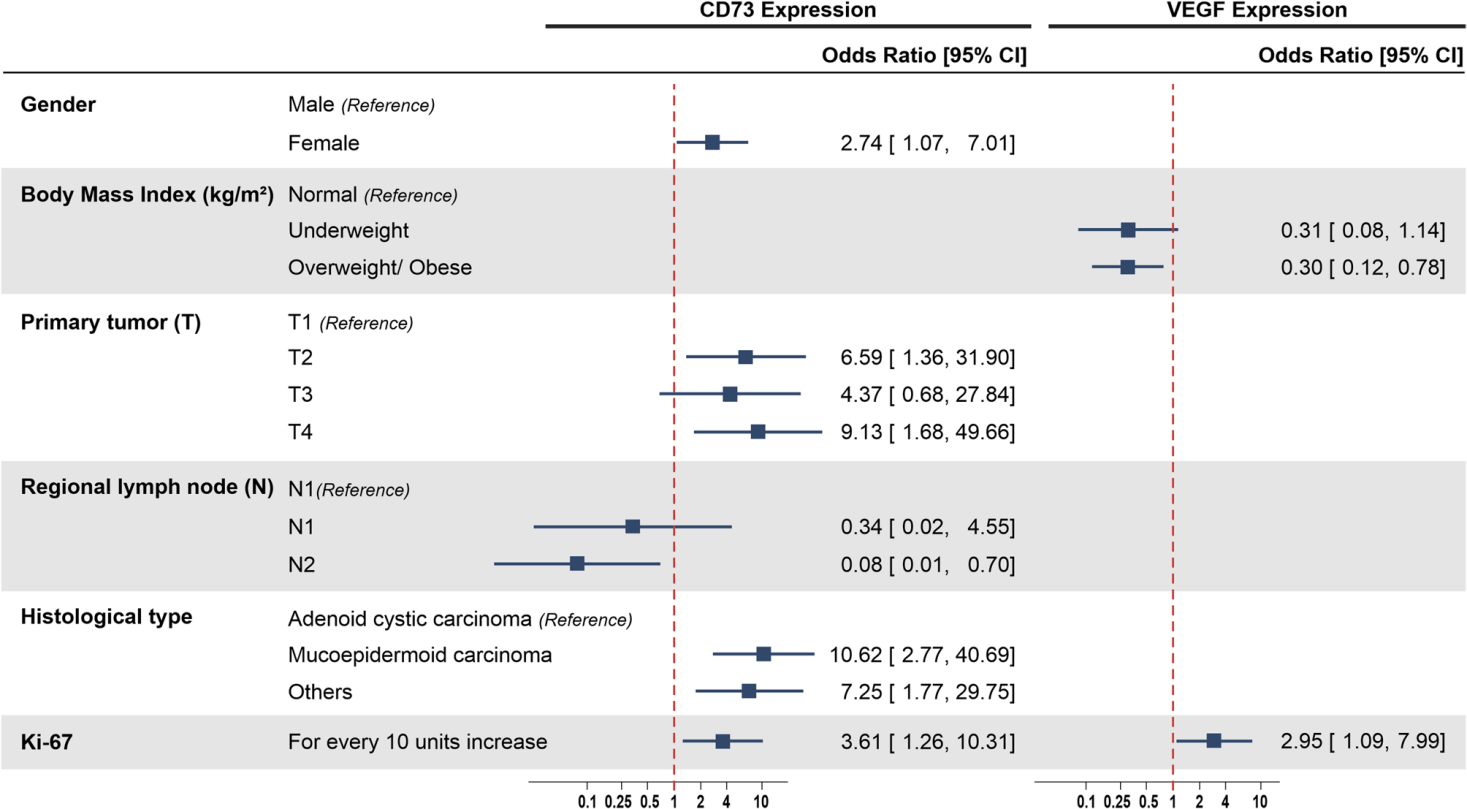
Clinicopathological factors independently associated with CD73 and VEGF expression

In the final multiple logistic regression, higher odds of having CD73 expression were found in female patients (OR = 2.74, 95% CI 1.07–7.01), those with higher level of primary tumor (T2: OR = 6.59, 95% CI 1.36–31.90; T4: OR = 9.13, 95% CI 1.68–49.66) and those with mucoepidermoid carcinoma (OR = 10.62, 95% CI 2.77–40.69). Moreover, for every increase of 10% in Ki-67 expression, the odds of having CD73 increased significantly (OR = 3.61, 95% CI 1.26–10.31). However, patients with regional lymph node level N2 were less likely to have CD73 expression (OR = 0.08, 95% CI 0.01–0.70) (Fig. 2). The model demonstrated good discriminatory power with an area under the ROC curve (AUC) of 0.80 (Appendix 3). Both the Pearson Chi-squared goodness-of-fit test (*p* = 0.514) and the Hosmer–Lemeshow test (*p* = 0.860) indicated that the model fits the data well.

For VEGF expression, BMI and Ki-67 were found to be independently associated with VEGF expression. Patients who were overweight/obese were less likely to have VEGF expression (OR = 0.30, 95% CI 0.12–0.78). In contrast, those who had a higher level of Ki-67 expression were more likely to have VEGF expression (OR = 2.95, 95% CI 1.09–7.99) (Fig. 2). The model showed acceptable discriminatory ability, with an area under the ROC curve (AUC) of 0.72 (Appendix 3). Model fit was supported by the Pearson Chi-squared test (*p* = 0.163) and the Hosmer–Lemeshow test (*p* = 0.299), indicating that the predicted probabilities were consistent with the observed outcomes.

## Discussion

The expression of CD73 and VEGF has shown potential as a prognostic biomarker and a target for therapeutic intervention in various types of cancer, including salivary gland carcinoma [21–23]. Understanding these two potential factors’ mechanisms of action and clinical applications could significantly improve treatment outcomes and give new strategies to manage this disease. Nevertheless, its practical application has not been systematically implemented in the SGC population, especially in Southeast Asian patients. Our study is the first in Vietnamese population with SGC and reveals a relatively high prevalence of expression of CD73 (53.2%) and VEGF (66.7%). The study also revealed important, independent associated factors of these expressions, including gender, BMI, primary tumor, regional lymph note, histological type and Ki-67.

The expression of CD73 in SGCs demonstrates significant variability across studies, underscoring its crucial role in immune evasion and tumor progression. For example, our study found CD73 expression in 53.2% of cases. In contrast, Ranjbar et al. (2019) reported a much higher expression rate of 97% in salivary gland tumors compared to only 3.6% in normal tissues (*P* < 0.001) [10]. Moreover, Bauer et al. (2023) provided a more detailed perspective, showing CD73 expression in 21.1% of tumor cells and 42.9% of immune cell infiltrates (TPS ≥ 1%), with the highest rates observed in specific subtypes such as MEC (30%), ACC (29%), and PLGA (33%) [8]. These variations underscore the influence of methodology, scoring systems, and tumor histology on reported expression rates, highlighting the universal upregulation of CD73 in malignancy while pointing to potential regional or methodological differences. Moreover, the high rate of VEGF expression in our study emphasizes its critical role in angiogenesis and tumor progression, consistent with the findings from other studies. For instance, Lequerica-Fernández et al. (2007) reported a VEGF expression rate of 62% [24]. Lee et al. (2012) observed a higher positivity rate of 88% with varying expression intensities in SGC cases [25]. Similarly, Bayat et al. (2023) demonstrated a direct association between VEGF levels and tumor aggressiveness in adenoid cystic carcinoma, with higher expression in advanced grades [12]. Dos Santos et al. (2021) reported VEGF expression in 87% of cases, linking it to angiogenesis and poor outcomes in certain studies, although this association was inconsistent [21].

Our study revealed a significant association between female gender and increased CD73 expression in SGCs, suggesting a considerable impact of gender on tumor biology and immune modulation. This finding diverges from Ranjbar et al. (2019), who reported no gender-based differences in CD73 expression. Such disparities may derive from variations in cohort features or the hormonal milieu [10]. Estrogen, a key regulator of immune responses, has been shown to influence CD73 expression in estrogen receptor-positive cancers like breast cancer [26, 27]. Elevated estrogen levels in women could potentially cause CD73 overexpression, promoting immune evasion and facilitating tumor progression. Moreover, women generally exhibit stronger immune responses than men, possibly creating a tumor microenvironment conducive to CD73 upregulation. Klein and Morgan (2020) highlighted the need to explore biological and sociocultural factors shaping male-female disparities in immune responses and treatment outcomes. Conversely, male-dominated immune profiles may suppress CD73 expression, reflecting distinct immune dynamics [28].

Our study also found that overweight or obese patients were significantly less likely to display VEGF expression in SGCs. This result contrasts with studies on other cancer types, where obesity is often related to increased VEGF expression. For example, previous studies found elevated VEGF levels in obese breast cancer patients, attributing this to chronic inflammation and adipose tissue-driven angiogenesis [29, 30]. Meanwhile, Dos Santos et al. (2021) found VEGF overexpression in salivary gland tumors correlating with aggressive tumor behavior, irrespective of BMI [21]. The discrepancy between our findings and international research may originate from population-specific factors, including genetic predispositions, dietary habits, and environmental exposures, which can uniquely affect the association between BMI and VEGF expression [31–33].

Our study identified a significant association between higher primary tumor stages (T2 and T4) and increased CD73 expression in SGCs. This finding aligns with existing literature, which suggests that elevated CD73 levels correlate with advanced tumor stages and may contribute to tumor progression through immunosuppressive mechanisms [22, 34]. Interestingly, while T4 stage did not show a significant association in the univariate analysis, it became significant after adjusting for other factors in the multivariate model. This suggests that the relationship between T4 and CD73 expression may be influenced by other characteristics and highlights the importance of considering these factors together. Additionally, we observed that patients with MEC exhibited higher odds of CD73 expression. This is consistent with previous research indicating that CD73 expression is significantly higher in MEC compared to other salivary gland tumor types [10]. Interestingly, our study found that patients with regional lymph node involvement at level N2 were less likely to express CD73. This contrasts with some studies that have reported higher CD73 expression in lymph node metastasizing cancers [10]. The discrepancy may be due to differences in sample size, tumor biology, or regional variations in patient populations.

Our study found that the increase in Ki-67 expression was associated with the significant increase in CD73 expression. This aligns with previous studies, such as the research by Zhang et al. (2010), which demonstrated that chronic lymphocytic leukemia (CLL) cells with the highest CD73 expression also exhibited elevated Ki-67 levels, suggesting a relationship between cell proliferation and CD73 expression [35]. Additionally, we observed that higher Ki-67 expression was independently associated with VEGF expression. This finding is consistent with the study by Al-Harris et al. (2008), which showed a strong correlation between VEGF overexpression and Ki-67 in breast cancer, highlighting a connection between cell proliferation and angiogenesis [36]. These results underscore the importance of Ki-67 as a marker of cell proliferation, associated with both CD73 and VEGF expression, thereby influencing tumor progression and treatment response [37].

This study has several limitations. First, the relatively small sample size of SGC patients limits the generalizability of the findings. Given the rarity of this cancer type, the study lacks sufficient statistical power to confirm potential associations between biomarkers and prognosis on a larger scale. Second, although our samples were obtained from the largest cancer-specialized hospital in southern Vietnam (i.e., Ho Chi Minh City Oncology Hospital), the single-center design limits the generalizability of our findings. Therefore, the observed characteristics of SGC in this study may not fully represent those of the wider Vietnamese population, particularly rural or diverse ethnic subgroups. Third, its cross-sectional design provides only a snapshot of the relationships between CD73, VEGF expression, and clinicopathological characteristics, making it impossible to establish causal relationships or assess the progression and treatment outcomes of the disease. Fourth, the absence of long-term clinical follow-up data restricts the study’s ability to evaluate the prognostic significance and clinical applicability of CD73 and VEGF in personalized treatment strategies. These limitations highlight the need for larger, longitudinal studies with extended follow-up to validate these findings and broaden their clinical relevance.

## Conclusion

The high expression rates of CD73 and VEGF found in our study highlight their important roles in salivary gland cancer. Several important, independent factors were also found and can be used to identify patients with a higher likelihood of having these expressions to support targetting high risk populations. These findings provide a foundation for integrating CD73 and VEGF expression into clinical decision-making, helping clinicians identify patients who may benefit from targeted therapies and guiding personalized treatment strategies for salivary gland carcinoma.

## Supplementary Information

Supplementary Material 1.

## Data Availability

The datasets used and/or analyzed during the current study are available from the corresponding author on reasonable request.

## Abbreviations

SGC: Salivary gland carcinoma
H&E: Hematoxylin and eosin
FFPE: Paraffin-embedded tissue blocks
MEC: Mucoepidermoid carcinoma

## References

[1] To VS, Chan JY, Tsang RK, Wei WI. Review of salivary gland neoplasms. ISRN Otolaryngol. 2012;2012:872982. 23724273 10.5402/2012/872982 PMC3658557

[2] Ettl T, Schwarz-Furlan S, Gosau M, Reichert TE. Salivary gland carcinomas. Oral Maxillofac Surg. 2012;16(3):267–83. 22842859 10.1007/s10006-012-0350-9

[3] Global Cancer Observatory. Cancer Today. https://gco.iarc.who.int/today. [Accessed 20 May 2024].

[4] Salivary Gland Neoplasms. http://emedicine.medscape.com/article/852373. [Accessed 16 May 2024].

[5] Lukovic J, Alfaraj FA, Mierzwa ML, Marta GN, Xu W, Su J, Moraes FY, Huang SH, Bratman SV, O’Sullivan B, et al. Development and validation of a clinical prediction-score model for distant metastases in major salivary gland carcinoma. Annals Oncology: Official J Eur Soc Med Oncol / ESMO. 2020;31(2):295–301. 10.1016/j.annonc.2019.10.024 31959347

[6] Ouyang DQ, Liang LZ, Zheng GS, Ke ZF, Weng DS, Yang WF, Su YX, Liao GQ. Risk factors and prognosis for salivary gland adenoid cystic carcinoma in Southern china: A 25-year retrospective study. Med (Baltim). 2017;96(5):e5964. 10.1097/MD.0000000000005964 PMC5293447 28151884

[7] Bach N, Winzer R, Tolosa E, Fiedler W, Brauneck F. The clinical significance of CD73 in cancer. Int J Mol Sci. 2023;24(14):1–21. 10.3390/ijms241411759 PMC10380759 37511518

[8] Bauer A, Gebauer N, Knief J, Tharun L, Arnold N, Riecke A, Steinestel K, Witte HM. The expression of the adenosine pathway markers CD39 and CD73 in salivary gland carcinomas harbors the potential for novel immune checkpoint Inhibition. J Cancer Res Clin Oncol. 2023;149(7):3193–208. 35902382 10.1007/s00432-022-04211-x PMC10314850

[9] Allard B, Turcotte M, Spring K, Pommey S, Royal I, Stagg J. Anti-CD73 therapy impairs tumor angiogenesis. Int J Cancer J Int Du Cancer. 2014;134(6):1466–73. 10.1002/ijc.28456 23982901

[10] Ranjbar MA, Ranjbar Z, Zahed M, Nikookar N. CD73 a novel marker for the diagnosis of benign and malignant salivary gland tumors. J Clin Experimental Dentistry. 2019;11(3):e213–8. 10.4317/jced.54918 PMC6461735 31001389

[11] Pouloudi D, Sotiriadis A, Theodorakidou M, Sarantis P, Pergaris A, Karamouzis MV, Theocharis S. The impact of angiogenesis in the most common salivary gland malignant tumors. Int J Mol Sci. 2020;21(24):1–17. 10.3390/ijms21249335 PMC7762607 33302367

[12] Bayat P, Mahdavi N, Younespour S, Kardouni Khoozestani N. Interactive role of miR-29, miR-93, miR-205, and VEGF in salivary adenoid cystic carcinoma. Clin Exp Dent Res. 2023;9(1):112–21. 36281584 10.1002/cre2.678 PMC9932236

[13] Micaily I, Johnson J, Argiris A. An update on angiogenesis targeting in head and neck squamous cell carcinoma. Cancers Head Neck. 2020;5(1):5. 32280512 10.1186/s41199-020-00051-9 PMC7132887

[14] Nguyen TKC, Nguyen TH, Tran VT. Clinical analysis of salivary gland tumor cases in 2009 and 2010. Vietnamese J Oncol. 2011;3:107–14.

[15] Nguyen VC. Histopathological study and immunohistochemical characteristics of salivary gland carcinoma. J Practical Med. 2012;838(8/2012):78–83.

[16] John KCC. WHO classification of head and neck tumours. 4th ed. International Agency for Research on Cancer; 2017.

[17] Ben-Izhak O, Akrish S, Nagler RM. Ki67 and salivary cancer. Cancer Invest. 2008;26(10):1015–23. 19093259 10.1080/07357900802088968

[18] Ettl T, Schwarz S, Kleinsasser N, Hartmann A, Reichert TE, Driemel O. Overexpression of EGFR and absence of C-KIT expression correlate with poor prognosis in salivary gland carcinomas. Histopathology. 2008;53(5):567–77. 18983466 10.1111/j.1365-2559.2008.03159.x

[19] Park S, Nam SJ, Keam B, Kim TM, Jeon YK, Lee SH, Hah JH, Kwon TK, Kim DW, Sung MW, et al. VEGF and Ki-67 overexpression in predicting poor overall survival in adenoid cystic carcinoma. Cancer Res Treat. 2016;48(2):518–26. 26194375 10.4143/crt.2015.093 PMC4843710

[20] Hosmer DWLS, Sturdivant RX. Applied logistic regression, Third edition edn. Wiley: Wiley series in probability and statistics; 2013.

[21] Dos Santos E, Ramos JC, Normando AG, Leme AF. Prognostic value of the immunohistochemical expression of vascular endothelial growth factors in malignant salivary gland neoplasms: a systematic review and meta-analysis. Med Oral Patologia Oral Y Cir Bucal. 2021;26(2):e126–35. 10.4317/medoral.23974 PMC7980300 33609023

[22] Xia C, Yin S, To KKW, Fu L. CD39/CD73/A2AR pathway and cancer immunotherapy. Mol Cancer. 2023;22(1):44. 36859386 10.1186/s12943-023-01733-x PMC9979453

[23] Jiang T, Xu X, Qiao M, Li X, Zhao C, Zhou F, Gao G, Wu F, Chen X, Su C, et al. Comprehensive evaluation of NT5E/CD73 expression and its prognostic significance in distinct types of cancers. BMC Cancer. 2018;18(1):267. 29514610 10.1186/s12885-018-4073-7 PMC5842577

[24] Lequerica-Fernandez P, Astudillo A, de Vicente JC. Expression of vascular endothelial growth factor in salivary gland carcinomas correlates with lymph node metastasis. Anticancer Res. 2007;27(5B):3661–6. 17972532

[25] Lee SK, Kwon MS, Lee YS, Choi S-H, Kim SY, Cho KJ, Nam SY. Prognostic value of expression of molecular markers in adenoid cystic cancer of the salivary glands compared with lymph node metastasis: a retrospective study. World J Surg Oncol. 2012;10(1):266. 23231994 10.1186/1477-7819-10-266 PMC3556129

[26] Katsuta E, Anand V, Yan L, Dasgupta S, Takabe K. Abstract 5200: high CD73 expression, regulated by Estrogen signaling, associates with cancer aggressiveness in Estrogen receptor (+) breast cancer. Cancer Res. 2019;79(13Supplement):5200–5200.

[27] Lee J, Park H, Moon S, Do JT, Hong K, Choi Y. Expression and regulation of CD73 during the estrous cycle in mouse uterus. Int J Mol Sci. 2021;22(17):1–12 . 10.3390/ijms22179403 PMC8431015 34502315

[28] Klein SL, Morgan R. The impact of sex and gender on immunotherapy outcomes. Biology Sex Differences. 2020;11(1):24. 10.1186/s13293-020-00301-y PMC7197158 32366281

[29] Hu C, Chen X, Yao C, Liu Y, Xu H, Zhou G, Xia H, Xia J. Body mass index-associated molecular characteristics involved in tumor immune and metabolic pathways. Cancer Metabolism. 2020;8(1):21. 32999719 10.1186/s40170-020-00225-6 PMC7517824

[30] Evangelista GCM, Salvador PA, Soares SMA, Barros LRC, Xavier FHC, Abdo LM, Gualberto ACM, Macedo GC, Clavijo-Salomon MA, Gameiro J. 4T1 mammary carcinoma colonization of metastatic niches is accelerated by obesity. Front Oncol. 2019;9:1–12. 10.3389/fonc.2019.00685 PMC6764084 31616626

[31] Chermon D, Birk R. Deciphering the interplay between genetic risk scores and lifestyle factors on individual obesity predisposition. Nutrients. 2024;16(9):1296. 38732542 10.3390/nu16091296 PMC11085817

[32] van Vliet-Ostaptchouk JV, Snieder H, Lagou V. Gene–Lifestyle interactions in obesity. Curr Nutr Rep. 2012;1(3):184–96. 24392269 10.1007/s13668-012-0022-2 PMC3873060

[33] Klibaner-Schiff E, Simonin EM, Akdis CA, Cheong A, Johnson MM, Karagas MR, Kirsh S, Kline O, Mazumdar M, Oken E, et al. Environmental exposures influence multigenerational epigenetic transmission. Clin Epigenetics. 2024;16(1):145. 39420431 10.1186/s13148-024-01762-3 PMC11487774

[34] Zhang B. CD73: A novel target for cancer immunotherapy. Cancer Res. 2010;70(16):6407–11. 20682793 10.1158/0008-5472.CAN-10-1544 PMC2922475

[35] Serra S, Horenstein AL, Vaisitti T, Brusa D, Rossi D, Laurenti L, D’Arena G, Coscia M, Tripodo C, Inghirami G, et al. CD73-generated extracellular adenosine in chronic lymphocytic leukemia creates local conditions counteracting drug-induced cell death. Blood. 2011;118(23):6141–52. 21998208 10.1182/blood-2011-08-374728 PMC3342854

[36] Al-Harris ES, Al-Janabi AA, Al-Toriahi KM, Yasseen AA. Over expression of vascular endothelial growth factor in correlation to Ki-67, grade, and stage of breast cancer. Saudi Med J. 2008;29(8):1099–104. 18690299

[37] Al-Harris ES, Al-Janabi AaA, Al-Toriahi KM, Yasseen AA. Over expression of vascular endothelial growth factor in correlation to Ki-67, grade, and stage of breast cancer. Saudi Med J. 2008;29(8):1099–104. 18690299

