# Supplementary material for: Expression of CD73 and VEGF in salivary gland carcinomas: associations with clinicopathological characteristics in Vietnamese population
Source: BMC Cancer. 2025 Oct 31;25:1678. doi: 10.1186/s12885-025-15129-1 (PMC12577387; doi:10.1186/s12885-025-15129-1)
Supplement: Supplementary file 1 — Supplementary Material 1. [file 12885_2025_15129_MOESM1_ESM.docx]

**Appendix 1:** VEGF and CD73 Expression (×200)


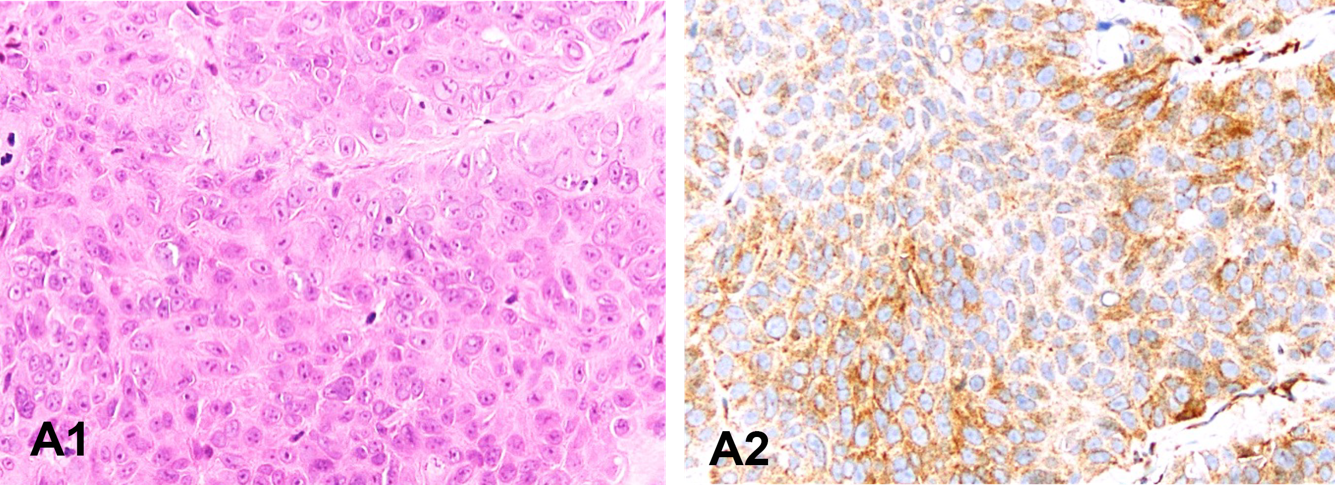

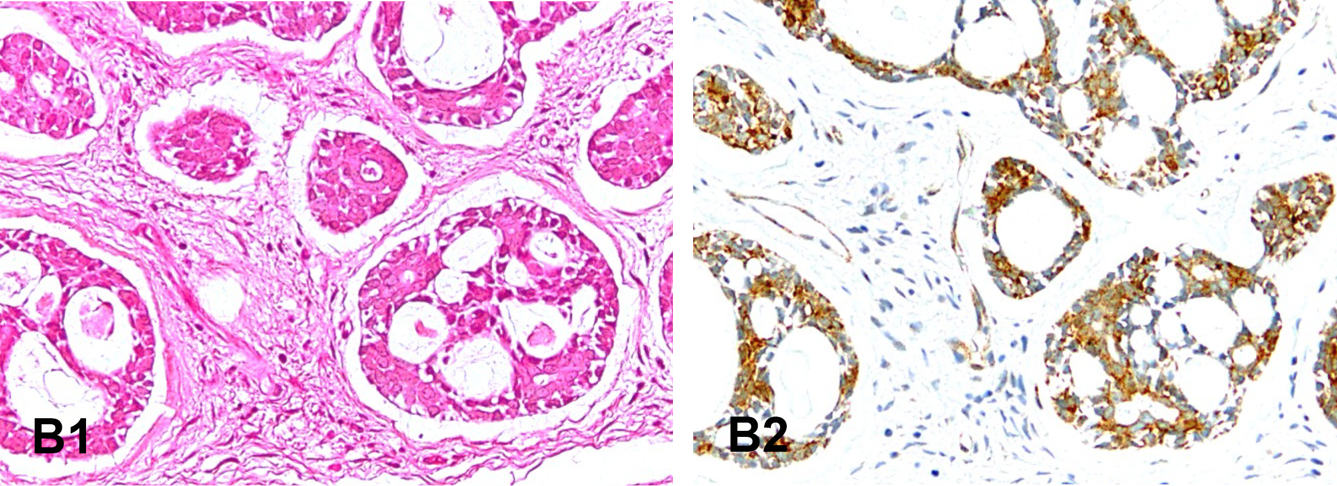

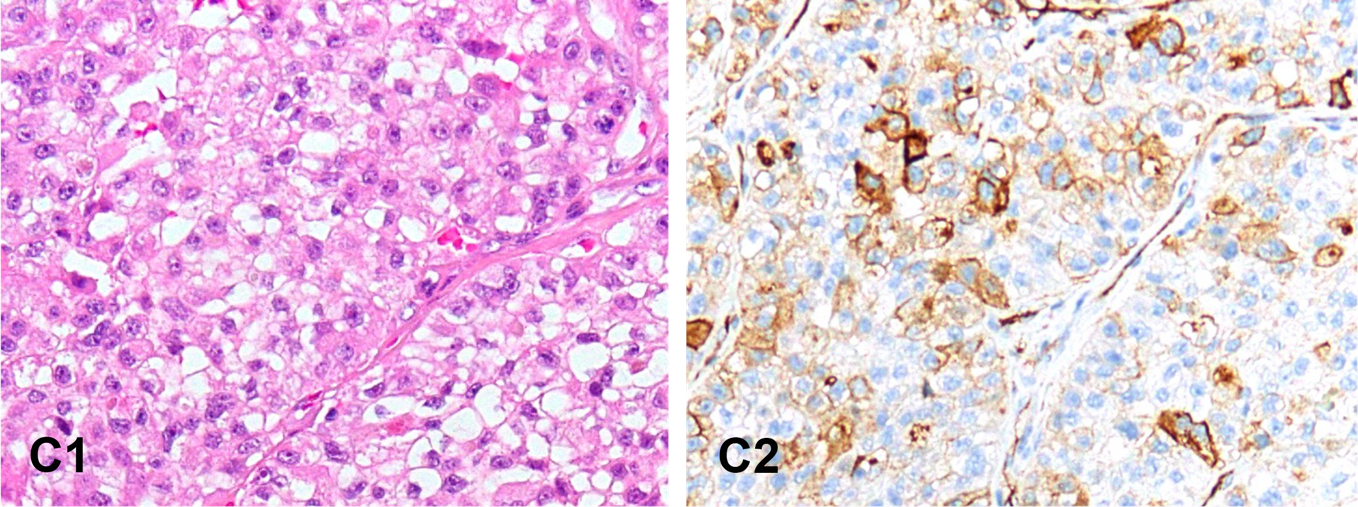

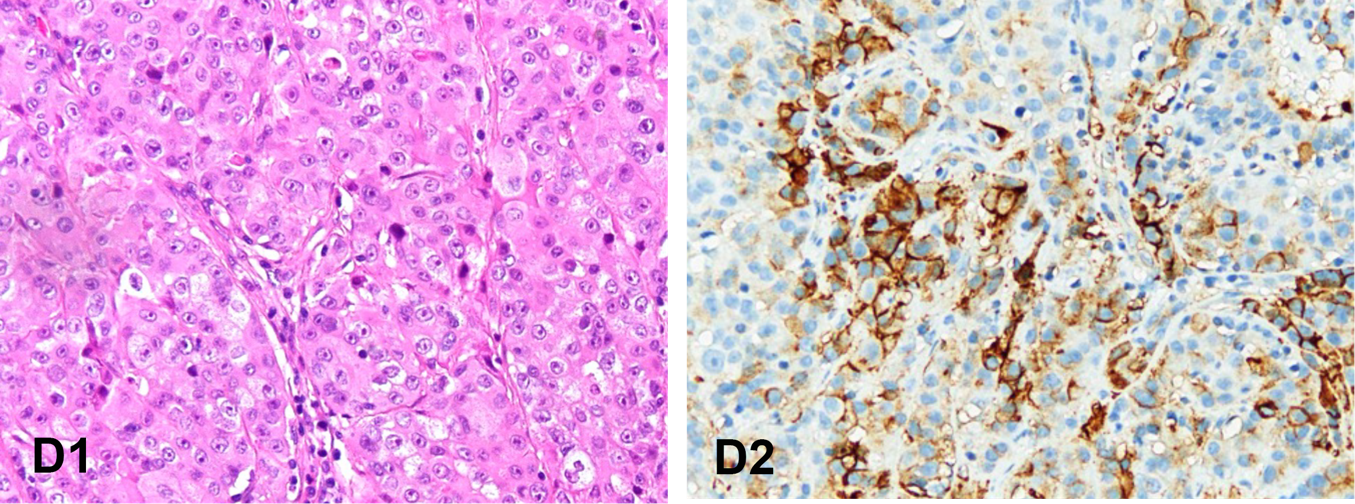


*A1. Mucoepidermoid carcinoma (HE); A2. Positive VEGF expression in mucoepidermoid carcinoma; B1. Adenoid cystic carcinoma (HE), B2. Positive VEGF expression in adenoid cystic carcinoma; C1. Mucoepidermoid carcinoma (HE); C2. Positive CD73 expression in mucoepidermoid carcinoma; D1. Acinic cell carcinoma (HE), D2. Positive CD73 expression in acinic cell carcinoma.*

**Appendix 2**: Histological types of salivary gland carcinoma

| **SGC** | **N**  *(n=111)* | **%** |
| --- | --- | --- |
| Mucoepidermoid carcinoma | 59 | 53.2 |
| Adenoid cystic carcinoma | 26 | 23.4 |
| Acinic cell carcinoma | 14 | 12.6 |
| Pleomorphic adenoma carcinoma | 6 | 5.4 |
| Basal cell adenocarcinoma | 3 | 2.7 |
| Epithelial-myoepithelial carcinoma | 2 | 1.8 |
| Carcinoma ex pleomorphic adenoma | 1 | 0.9 |

**Appendix 3:** Model performance for multivariable logistic regression models predicting CD73 and VEGF expression

| **CD73 expression**    Model performance: p = 0.514 (Pearson), p = 0.860 (Hosmer–Lemeshow) |
| --- |
| **VEGF expression**    Model performance: p = 0.163 (Pearson), p = 0.299 (Hosmer–Lemeshow) |
